# Evaluation of DEM Accuracy Improvement Methods Based on Multi-Source Data Fusion in Typical Gully Areas of Loess Plateau

**DOI:** 10.3390/s23083878

**Published:** 2023-04-11

**Authors:** Jin Huang, Lan Wei, Tao Chen, Mingliang Luo, Hui Yang, Yunyun Sang

**Affiliations:** 1 School of Geographical Sciences, China West Normal University, Nanchong 637009, China; 2 Sichuan Provincial Engineering Laboratory of Monitoring and Control for Soil Erosion on Dry Valleys, China West Normal University, Nanchong 637009, China

**Keywords:** elevation RMSE, Gram–Schmidt pan sharpening, feature points embedding, weight fusion, DEM

## Abstract

Improving the accuracy of DEMs is a critical goal in digital terrain analysis. The combination of multi-source data can be used to increase DEM accuracy. Five typical geomorphic study areas in the Loess Plateau in Shaanxi were selected for a case study and a 5 m DEM unit was used as the basic data input. Data from three open-source databases of DEM images, the ALOS, SRTM and ASTER, were obtained and processed uniformly through a previously geographical registration process. Three methods, Gram–Schmidt pan sharpening (GS), weighted fusion and feature-point-embedding fusion, were used for mutual enhancement of the three kinds of data. We combined the effect of these three fusion methods in the five sample areas and compared the eigenvalues taken before and after the fusion. The main conclusions are as follows: (1) The GS fusion method is convenient and simple, and the three combined fusion methods can be improved. Generally speaking, the fusion of ALOS and SRTM data led to the best performance, but was greatly affected by the original data. (2) By embedding feature points into three publicly available types of DEM data, the errors and extreme error value of the data obtained through fusion were significantly improved. Overall, ALOS fusion resulted in the best performance because it had the best raw data quality. The original eigenvalues of the ASTER were all inferior and the improvement in the error and the error extreme value after fusion was evident. (3) By dividing the sample area into different areas and fusing them separately according to the weights of each area, the accuracy of the data obtained was significantly improved. In comparing the improvement in accuracy in each region, it was observed that the fusion of ALOS and SRTM data relies on a gentle area. A high accuracy of these two data will lead to a better fusion. Merging ALOS and ASTER data led to the greatest increase in accuracy, especially in the areas with a steep slope. Additionally, when SRTM and ASTER data were merged, the observed improvement was relatively stable with little difference.

## 1. Introduction

The Loess Plateau is the largest loess area in the world. The Loess Plateau is an active area of neotectonic movement, which is mainly characterized by the intermittent uplift of large areas in the plateau and the continuous subsidence of the surrounding down-depression area. It is generally believed that the Loess Plateau is formed on the basis of loess underlying the bedrock. The primary loess is the aeolian dust accumulation under the dry and cold climate of quaternary glaciation and the secondary loess is the primary loess transformed by diluvial and alluvial processes. During the quaternary loess accumulation period, the loess stratum showed the alternations of loess and paleosol with the climatic cycles of glacial and interglacial periods. This unique landform developed under the action of internal and external forces such as water and heat, making it one of the most valuable regions for geosensitive research in the world. With the continuous improvement in the methods of digital terrain technology, the complex and unique terrain of the Loess Plateau also provides a unique sample for the in-depth study of digital terrain analysis [1]. The publicly available digital elevation model (DEM) provides the necessary data basis for digital topography research of the Loess Plateau. However, the quality and accuracy of publicly available DEM data are limited to a certain extent due to the influence of their imaging principles and production technologies. The development of digital terrain research depends on the continuous improvement in DEM accuracy. Although evolving observational techniques offer the possibility of acquiring high-accuracy DEM data products, new data acquisition is costly, time-consuming and still inevitably limited by observational techniques and surface environments. Multi-source data fusion is another effective way to improve data quality. Therefore, it is of great significance to explore higher-precision DEM data based on publicly available DEM data for digital terrain research on the Loess Plateau [2].

Scholars at home and abroad have conducted various experiments to improve the accuracy of DEM. Bandara, Yue et al. filled the SRTM gap via the DSF filling method and optimized the TIN filling method, using this method to integrate various data to improve accuracy [3,4]. Gallant and O’Loughlin et al. aimed to improve DEM accuracy by removing vegetation errors [5,6]. From the perspective of mathematical analysis, Karakasis et al. used binary function to expand DEM data and carried out weighted fusion processing on the coefficient matrix obtained from the expansion [7]. Siart, Yamazaki and Kaab attempted to integrate a global DEM with a high-precision DEM [8,9]. Podobnikar and Papasaika proposed weight fusion and DEM sparse point general fusion methods [10,11]. Hung T. et al. used a weighted linear combination method to fuse data to improve DEM accuracy [12]. Sun Liang et al. proposed adopting an algorithm for calculating the optimal weighted fusion DEM in the spatial domain through experiments [13]. Huang Changjun introduced Shepard’s weighted model to improve the fitting of moving surfaces. Zhao Mingwei optimized the directionality and distribution of sampling points from the perspective of original data by locally calculating the coordinate rotation of the region and adding constraint conditions and calculated the relationship between grid dots and elevation to reduce errors [14,15]. Fan Jieming proposed a new interpolation model based on an irregular grid, Wang Ketao proposed a weighted average combination method of double higher-order polynomials and Du Manfei et al. proposed a variety of methods, such as interpolation, weighted average and combination, to study combination possibilities and find a method suitable for this region [16,17].

Based on research results at home and abroad, a multi-source data fusion algorithm has been proved to be able to improve the accuracy of the DEM. The acquisition of multi-source data is simple and the DEM can update quickly. Through the mutual enhancement of publicly available data, the demand for high precision and timeliness can be taken into account at the same time. However, although multi-source DEM data allow for a rich and diverse fusion method, the combination of different types of data for different fusion methods needed to obtain specific results may vary; using the same combination of data in the same fusion method corresponding to different types of topography effects will lead to different results. However, no domestic or foreign scholars have carried out an analysis of this. Therefore, based on three publicly available data sources, the ALOS, SRTM and ASTER, this study adopted three methods, namely, GS fusion, feature-point-embedding fusion and regional weight fusion. Taking typical geomorphic regions of the Loess Plateau in northern Shaanxi as samples, the mutual enhancement of fusion was conducted to reduce DEM errors and improve accuracy. We explored the accuracy improvement effect of the three research methods, including which data combination has the best accuracy effect and the best degree of improvement, and which combinations and methods are suitable for each landform. The research results provide a reference for algorithms and strategies aiming to improve the accuracy of DEM on the Loess Plateau and their regional applicability.

## 2. Materials and Methods

### 2.1. Study Areas

The Loess Plateau water system with the Yellow River as the backbone. There are about 200 rivers that originated in the Loess Plateau, including the larger rivers: Taohe River, Zuli River, Qingshui River, Huangfu River, Kuye River, Wuding River, Beiluo River, Wei River, Qin River, Fen River; i.e., the gullies are widely distributed on the loess hilly and gully region with a gully density of 4~8 km/km^2^. Due to the cutting and erosion of gullies and rivers, the Loess Plateau is characterized by numerous gullies and ravines.

**Figure 1:**
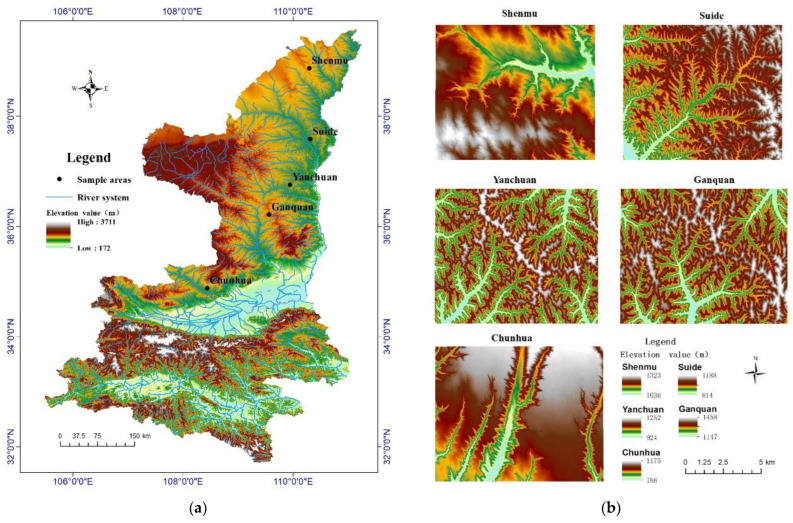
(**a**) The distribution of study areas; (**b**) hill shading of study areas.

The sample area in this article was selected from the Loess Plateau area in Shaanxi Province, specifically northern Shaanxi Province, which is at the center of the Loess Plateau. The geomorphologic landforms here are rich and diverse, with a vast area of approximately 12.4 square kilometers. The terrain is generally high in the northwest and low in the southeast. From the southern Weihe terraces to the north, there are beam-like hills, hilly hills and sandy loess landforms in the far north. In this study, five typical sample areas, Shenmu, Suide, Yanchuan, Ganquan and Chunhua, were selected from north to south to represent the topographic and geomorphic characteristics of different areas in the typical gully region of the Loess Plateau in northern Shaanxi. Among them, the Shenmu and Chunhua sample areas had sparse gully density, while the other three sample areas had dense gully distribution and complex topographic fluctuation. The distribution of the sample areas and their locations are shown in Figure 1 and the topographic and geomorphic features of the five sample areas are shown in Table 1.

### 2.2. Datasets

The experimental data in this research area are based on 1:10,000 (5 m resolution) basic data standardized by the national surveying and mapping department and three types of multi-source DEM data, SRTM, ASTER and ALOS data, which were publicly downloaded.

The full name of the ALOS is the advanced land observing satellite, an earth observation satellite launched by Japan in January 2006. It is equipped with an optical sensor, the panchromatic remote-sensing stereo mapping instrument (PRISM). PRISM takes observations between 82° N and 82° S latitude with a resolution of approximately 2.5 m; and, thus, has obvious advantages in digital elevation surveying and mapping.

The full name of the SRTM is the Shuttle Radar Topography Mission. In February 2000, this joint survey was completed by NASA and NIMA, as well as the German and Italian space agencies. The image data range includes 60° N–60° S, with a total area of over 119 million square kilometers, covering more than 80% of the Earth’s surface. The data are referenced by the WGS84 (World Geodetic System 1984), and the absolute plane accuracy and elevation accuracy are approximately ±20 m and ±16 m. These data types have a wide range of applications [18,19]. This study used SRTM1 data.

The full name of the ASTER is the advanced spaceborne thermal emission and reflected radiation imager. Data from ASTER were obtained from the TERRA Earth observation satellite launched by Japan and the United States (NASA) in December 1999. It uses stereo pair processing, and the spatial reference is consistent with SRTM, with a resolution of 30 m and an elevation accuracy of approximately ±20 m, covering 83° north–south latitude and up to 99% of the global land area [20].

**Figure 2:**
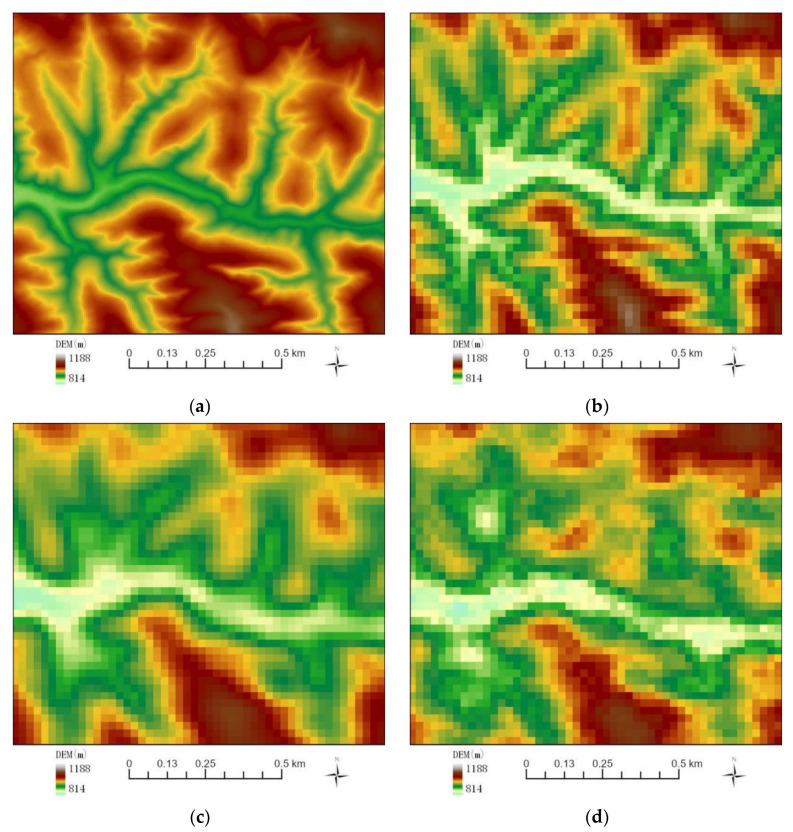
A comparison of four kinds of DEM data: (**a**) 5 m DEM; (**b**) ALOS DEM; (**c**) SRTM DEM; (**d**) ASTER DEM.

The 5 m DEM data were converted into the WGS84 (World Geodetic System 1984) coordinate system. The data were matched with SRTM data and other data, and the elevation was corrected by referencing the 0.35 m applicable value of elevation datum deviation correction in the research area. Then, the global fitting method was used to align the basic data with three grids to the east and two grids to the south. Finally, the spatial position and coordinate reference system of basic data and other data in the research area were unified [21]. The data characteristics are shown in Table 2 and a comparison of four kinds of DEM data is shown in Figure 2.

### 2.3. Methodology

**Figure 3:**
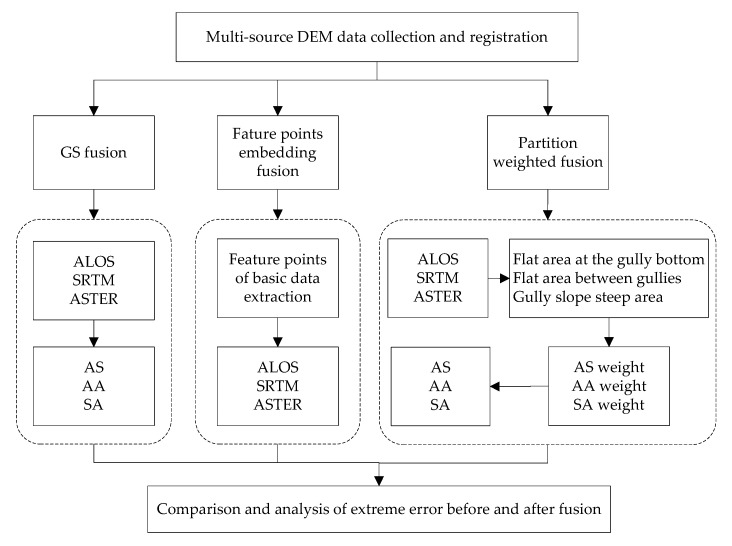
The flowchart of the research process.

This study selected five typical geomorphic sample regions in the Loess Plateau area as the research samples, including 5 m DEM data as basic data and the ALOS, SRTM and ASTER as three different data sources. Premenstrual registration processing, GS fusion, feature-point-embedding fusion and partition-weighted fusion were used for mutual enhancement. Using the three fusion methods for the five sample areas, the root mean squared error (RMSE) of elevation and the extreme RMSE before and after the fusion was carried out were compared to determine the optimal algorithm for improving DEM accuracy. The technical route is shown in Figure 3. AS represents the fusion result of ALOS and SRTM data, AA represents the fusion result of ALOS and ASTER data, and SA represents the fusion result of SRTM and ASTER data.

#### 2.3.1. Multi-Source Data Fusion Based on GS Transform

The GS transform fusion method can efficiently fuse and maximize the high-spatial-resolution image information contained in the fusion data, so that multi-source data can be efficiently and quickly fused. Firstly, the low-resolution image data were used to simulate the high-resolution image and this was used as the first component of GS fusion in GS fusion transformation with the low-resolution image. The formula for this process is as follows:

(1) $$GS_{T}\left(i,j\right)=(B_{T}\left(i,j\right)-\mu_{T})-\sum_{l=1}^{T-1}\left(\varphi \left(B_{T},GS_{l}\right)\times GS_{l}\left(i,j\right)\right)\;$$

(2) $$\mu_{T}=(\sum_{j=1}^{C}\sum_{i=1}^{R}(B_{T}\left(i,j\right))/C\times R$$

(3) $$\varphi \left(B_{T},GS_{l}\right)=\left[\frac{\sigma \left(B_{T},GS_{l}\right)}{\sigma {\left(GS_{l},GS_{l}\right)}^{2}}\right]\;$$

(4) $$\sigma_{T}=\sqrt{\frac{\sum_{j=1}^{cC}\sum_{i=1}^{R}\left(B_{T}\left(i,j\right)-\mu_{T}\right)}{C\times R}}\;$$

In the above formula, $GS_{T}$ represents the t-th component generated by GS transformation, and $B_{T}$ and $\mu_{T}$ represent the t-band image of the original image and the average gray value of the image (Equation (1)). $\varphi \left(B_{T},GS_{l}\right)$ represents the covariance between the T-band and $GS_{l}$ of the low-resolution image and $\sigma_{T}$ is used to calculate the standard deviation of $GS_{1}$ after the first transformation. *i*, *j* and *C*, *R* represent the number of rows and columns of low-resolution images and the whole remote sensing image, respectively.

According to the results of Equations (2)–(4), the high-resolution image was matched and optimized with the transformed results to obtain the optimized high-resolution image and the first component $GS_{1}$ was replaced. Then, the obtained data were changed using inverse GS to obtain the spatial-resolution-enhanced image results. Inverse transformation is represented by Equation (5):

(5) $${\hat{B}}_{T}\left(i,j\right)=GS_{T}\left(i,j\right)+\mu_{T}+\overset{T-1}{\underset{l=1}{\sum }}\varphi (B_{T},GS_{l})\times GS_{l}i,j\;$$

#### 2.3.2. Embedding Fusion Based on Feature Points

Ridges and valleys form the dividing lines (skeleton lines) of the topographic relief; so, they are of great importance for the study of topography and geomorphology. Ridges and valleys may be affected by factors such as shading and vegetation, which may lead to relatively large error values. In this case, the 5 m DEM basic high-precision data were used to extract the basic high-precision topographic feature points such as ridge and valley lines, which can be embedded into different data sources to improve the data accuracy.

The ridges and valleys represent water separation and catchment respectively. The extraction of ridge and valley lines is essentially the extraction of water-separation lines and catchment lines, so the extraction can be performed by using hydrological analysis.

**Figure 4:**
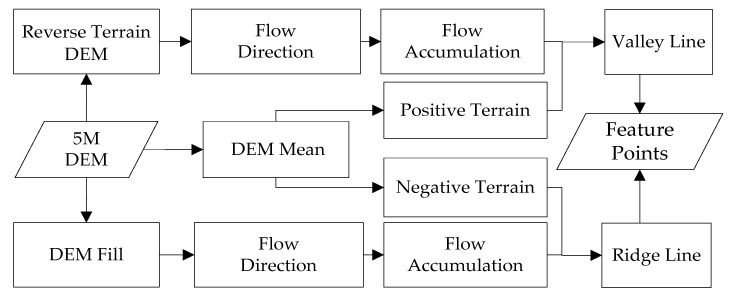
Feature points extraction flowchart.

For ridge lines, since it is also a water separation, the essence of the water separation is the origin of the flow. After the surface runoff simulation, the flow direction of these grids should only have the outflow direction, but not the flow direction; i.e., the accumulation of confluence in the DEM grids is zero. Therefore, by extracting the zero value, the divergence lines, i.e., the ridge lines, can be obtained. For valley lines, a reverse terrain calculation can be used. That is, a larger value is used to subtract the original DEM data to obtain the topographic data, which is opposite to the original DEM topography, so that the ridges in the original DEM become valleys in the reverse topography and the valleys in the original DEM become ridges in the reverse topography. Then, the extraction of valley lines can be achieved by using the ridge lines extraction method. The extraction method of feature points is shown in Figure 4.

#### 2.3.3. Multi-Source Data Fusion Based on Weight

**Figure 5:**
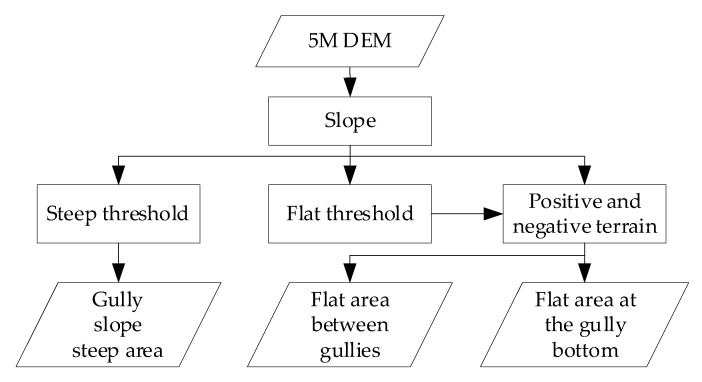
The study area division extraction flowchart.

The weight of image fusion was calculated according to the error of the original data, and then the original data were fused according to the weight. When the topography of the study area is complex and the data differ greatly, the error will be increased to some extent due to the influence of topography. Therefore, the samples were divided into gentle areas between gullies, gentle areas at the bottom of gullies and steep areas on gully slopes; and their weights were calculated for fusion. Figure 5 shows the extraction process of the research region.

The slope was calculated based on the basic data of 5 m DEM to obtain the gentle area at the bottom of the gully and the gentle area between the gullies in this area [22]. The elevation RMSE and positive and negative deviation of each sample area were calculated. Equation (6) was used to calculate the weight of different data in each area during data fusion. Finally, data from different sources were fused according to weight fusion Equation (7):

(6) $$w=\frac{\sigma_{2}{}^{2}-\rho \sigma_{1}\sigma_{2}}{\sigma_{1}{}^{2}+\sigma_{2}{}^{2}-2\rho \sigma_{1}\sigma_{2}}\;$$

(7) $$y_{c}=wf_{1}+(1-w)f_{2}$$

In the above formula, w represents the weight of fusion, which is generally between 0 and 1. $\sigma_{1}$ and $\sigma_{2}$ are the standard deviations of the fusion images, and ρ is the correlation coefficient between the image errors.$\;\;f_{1}$ and $f_{2}$ represent the original data before fusion.

Table 3 shows the best fusion weight of different partitions in various zones. The weights of AS, AA and SA in the table represent the weights of the ALOS, ALOS and SRTM, respectively, which are the first data sources used in the fusion process. The proportion of SRTM, ASTER and ASTER data in the second step is the difference between 1 and the proportion of the previous data. In some data, the error is obviously dominant and the fusion ratio is particularly close to 1; thus, it is represented by 1.

## 3. Results

Compared with the original data, the three fusion methods led to a certain improvement in data accuracy and they were able to better maintain the original advantages of the data and further optimize them to obtain the best results. This study was based on the 1:50,000 DEM accuracy standard of the National Bureau of Surveying and Mapping (Table 4) to analyze the degree of accuracy improvement of various fusion methods.

### 3.1. Multi-Source Data Fusion Based on GS Transform

The ALOS, SRTM and ASTER data of the Shenmu, Chunhua, Suide, Yanchuan and Suide plots were fused via GS transformation to obtain the error characteristic value results, as shown in Table 5.

According to the 1:50,000 accuracy standard of the National Bureau of Surveying and Mapping, the average slope of the Shenmu sample area before fusion is 8.75°, the error value in the SRTM data is between the reference value of the error in the flat and hilly land, and ALOS and ASTER data values fall between the hilly and mountain values. After the fusion, the AS data were improved by one level relative to the ALOS data and the accuracy of the AA and SA results did not improve by a whole level, but there was a significant improvement. The average slope of the Chunhua sample area was 11.96°. The first fusion combination of three kinds of data had values falling between the mountain and alpine mountain error reference values, though the latter were slightly larger. The accuracy level was not significantly improved by fusion, but the relative data effect was relatively poor. The maximum accuracy was increased by approximately 6.300 m, which is approaching the critical value of high accuracy. The average slopes of Ganquan, Suide and Yanchuan were all greater than 25°. The ALOS effect of Ganquan and Suide was relatively good (8.123, 9.958), falling within the error reference value range of 7 m–11 m in hilly and mountain. The error in SRTM and ASTER data fell between the value obtained for mountain and alpine mountain. After the fusion, the AS and AA results of these two plots were within the error reference value interval in the hilly and mountain. Compared with the SRTM and ASTER data, the accuracy level was improved and the error in the SA data was not significantly improved. The Yanchuan area has an average slope of 30.8° and the errors in the first combination of three kinds of data fell between the mountain and alpine mountain values. The accuracy improved significantly after the fusion process, but none of them improved by a whole level.

### 3.2. Embedding Fusion Based on Feature Points

**Figure 6:**
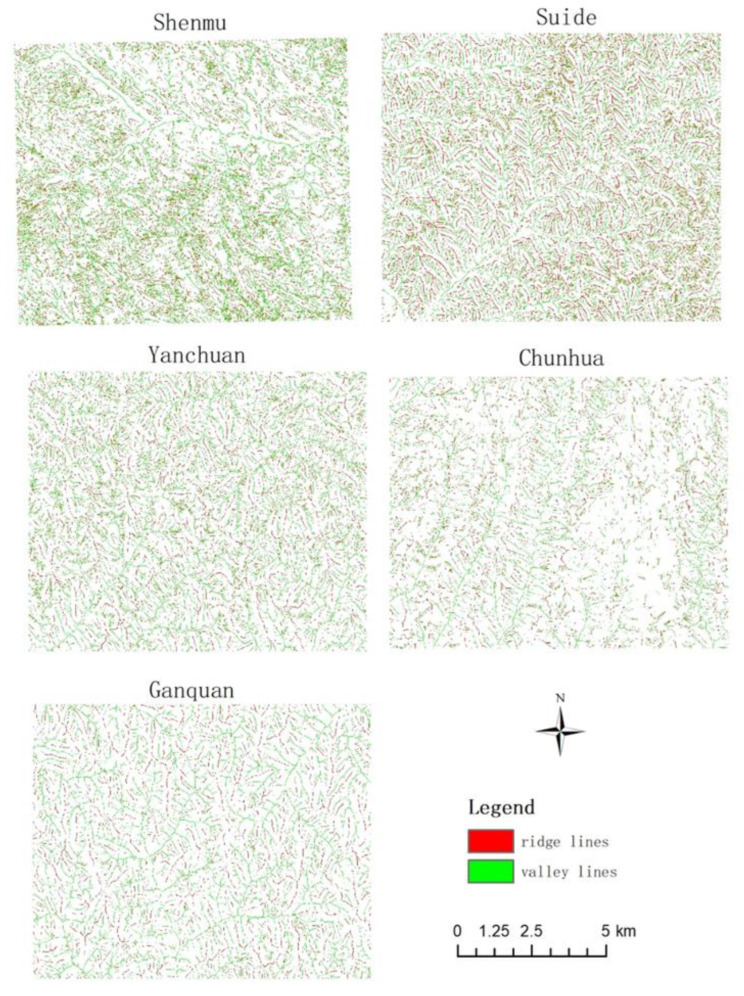
Valleys and ridges in study areas.

The results of feature point extraction are shown in Figure 6.

The feature points were embedded in different data and the results of the extreme error before and after fusion were tested and compared, as shown in Table 6.

According to the national standard of 1:50,000 DEM elevation error, the elevation RMSE value before Shenmu fusion generally fell between the hilly and mountain error value and the elevation RMSE value after fusion was less than 7, which is within the reference value range of 4–7 for flat land and hilly errors. The accuracy improvement was better. The lowest ALOS data value of the elevation RMSE before Suide fusion was 9.958 m, the largest ASTER elevation RMSE was 16.238 m and the maximum elevation RMSE after fusion was 9.507 m. Before fusion, ALOS data were close to the mountain error value and the other two fell between the mountain and alpine mountain values. Between 11 and 19 m, the elevation RMSE value after fusion fell between 7 and 11 m, and the accuracy of SRTM and ASTER data was improved by one level.

The Yanchuan sample area has the largest topographic undulations and the highest average slope. The elevation RMSE value corresponded to 11–19 m between mountain and alpine mountain. After the fusion of the three kinds of data, ALOS data has the RMSE value of 11.301 m, which is slightly higher than the mountain elevation RMSE value, but significantly reduced. This may have been greatly affected by the original data error; SRTM and ASTER values were lower than 11 m and the accuracy was improved by one level. The effect was obvious.

The error value of the data before the fusion of Chunhua was greater than the mountain error value standard and even the ASTER value was greater than that of the mountain area. After the fusion, the error value was reduced to meet the requirements of the next accuracy value standard interval. ALOS and SRTM values fell between the values of the hilly and mountain. The elevation RMSE reference value was between 7 and 11 m, and the ASTER value was between the mountain and alpine mountain reference value range of 11–19 m.

In the Ganquan sample area, the ALOS error value fell between 7 and 11 m in standard hilly and mountain, and SRTM and ASTER values were between 11 and 19 m in mountain and alpine mountain. After the feature points were embedded to improve the accuracy, the result values of these data all fell between 7 and 11 m, and the corresponding accuracy standard was between the hilly and mountain values.

The overall accuracy improvement effect achieved through feature embedding was evident, having been improved by one level, although some accuracy values were not improved by a whole level, and the elevation RMSE was also significantly reduced to close to the critical value.

### 3.3. Multi-Source Data Fusion Based on Weight

The error characteristic values before and after the weighted fusion of each plot are shown in Table 7, Table 8, Table 9, Table 10 and Table 11.

According to the 1:50,000 DEM quality standard of the State Bureau of Surveying and Mapping, the average slope of the Shenmu sample area is 8.75°, and the largest error between the flat area at the gully bottom and the flat area at the bottom of the original data is 8.111 m in the ASTER data, which falls between the values for the hilly and mountain. After fusion of weights, the maximum values were 5.272 m and 4.790 m, respectively, which fall between the reference value of the error in the elevation of flat land and hilly. The accuracy was increased by one level and the effect was improved; the error value before and after the fusion of steep areas fell between hilly and mountain values. It is closer to the error value of the hilly after fusion.

The maximum RMSE in the gentle area of Chunhua area are 18.473 m and 18.482 m, which fall between 11 and 19 m in mountain and alpine mountain values. The error value was significantly reduced through fusion, falling between that of hilly and mountain, and the accuracy was improved by one level. The steep area was similar to Shenmu. While the accuracy level was not significantly improved, the overall error value tended toward the mountain error value.

The topography of Ganquan, Suide and Yanchuan has large ups and downs, with average slopes of 26.42°, 28.55° and 30.8°, respectively, which generally correspond to the quality requirements of standard mountain and alpine mountain. The maximum values of the Ganquan flat area are 17.143 m and 11.430 m in mountain and alpine mountain; the maximum values after fusion were 14.593 m and 8.857 m. Although the error value of the flat area at the bottom of the ditch was larger than that of the mountain, it was optimized to the mountain value and the accuracy level of the flat area was increased to the range of error values from hilly to mountain. The error level in the steep area was not improved, but the elevation RMSE value was significantly reduced. Before the fusion of Suide, the elevation RMSE fell between 11 and 19 m in mountain and alpine mountain; in addition, after fusion, most of these values were lower than the mountain index value of 11 m, falling between the values for hilly and mountain, and the maximum RMSE remained between mountain and alpine mountain values. However, the decrease was evident, tending toward the mountain index value. The average slope of Yanchuan was the largest. Before the fusion, the elevation RMSE value was close to approximately 19 m in the alpine region. After the fusion, the elevation RMSE significantly decreased to close to the mountain index value of 11 m. In general, it can be concluded that the accuracy of the results was increased by one level or decreased significantly closer to the lower index value after fusion in a flat area. The accuracy in steep areas did not increase by a level, but it was close to the low index value.

## 4. Discussion

**Figure 7:**
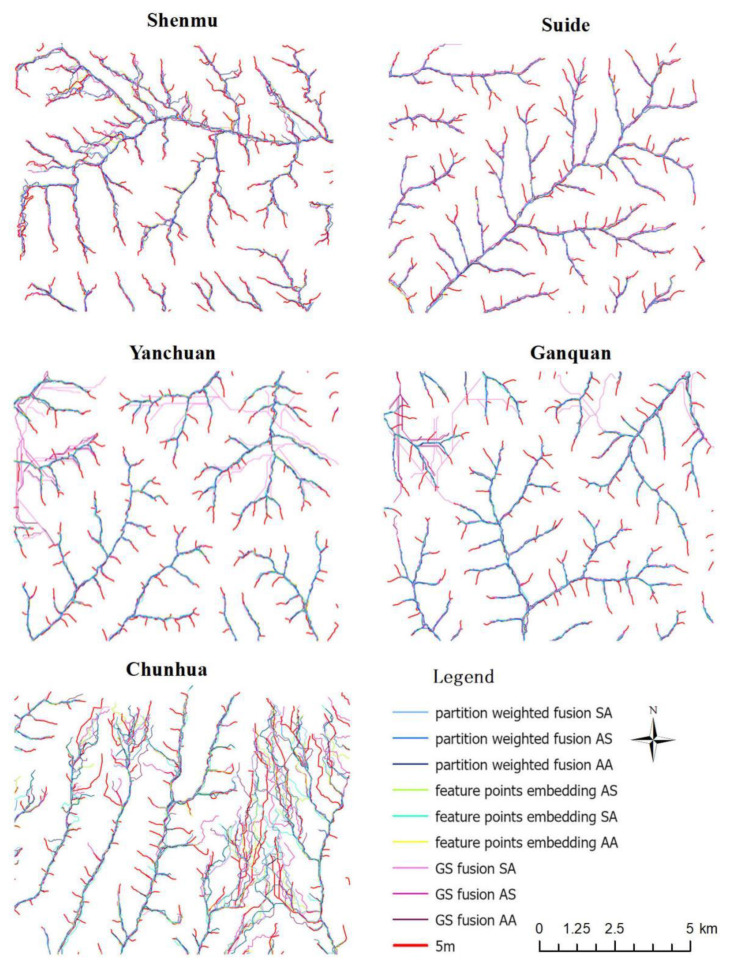
Comparison of river networks extracted by the three fusion methods for three types of DEM data and the river network extracted by 5 m DEM in the five study areas.

**Figure 8:**
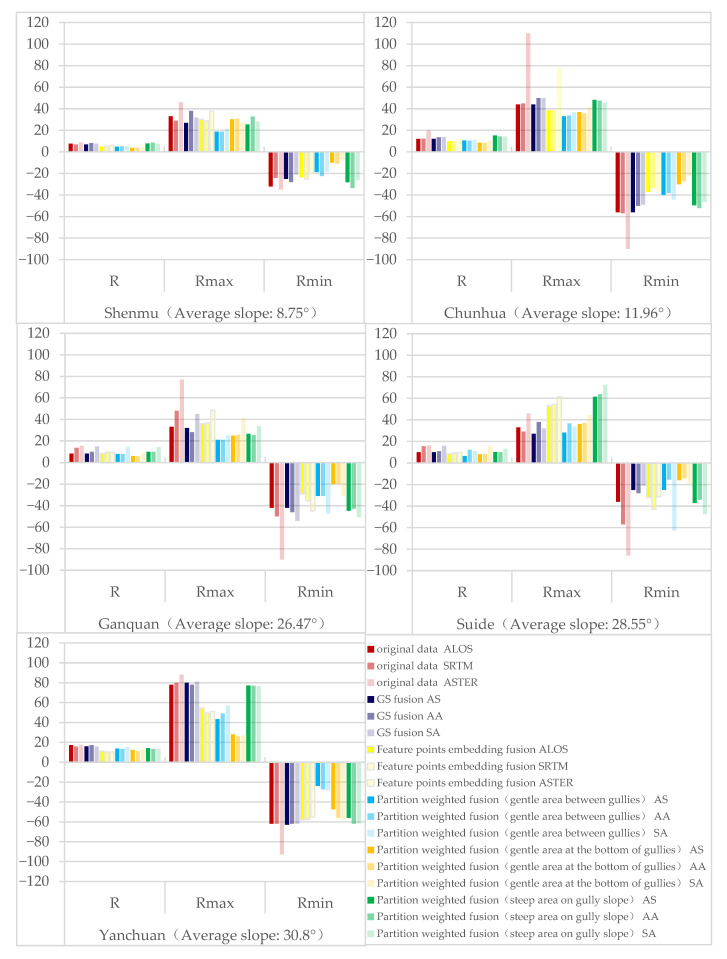
Error statistics before and after fusion.

Three multi-source DEM databases, the ALOS, SRTM and ASTER, were used in this study. The ALOS and ASTER are classified as optical remote sensing stereo mapping technology [23]. Observing the same area from different directions to obtain a stereo image pair will inevitably lead to shadows on the image, resulting in invalid values and limited accuracy. In addition, optical images are susceptible to weather and imaging conditions (such as clouds, haze, sunlight, etc.) This will affect the accuracy of the subsequent generated 3D elevation information [24]. ALOS–PRISM has a spatial resolution of 2.5 m, which is much higher than that of the ASTER at 30 m, and its strong stereoscopic observation capability gives it an unparalleled advantage over other satellites in digital elevation mapping [25]. The accuracy of the stereo pair extraction DEM is better in flat areas than in complex areas [26]. SRTM data are obtained by interferometric aperture radar [27]. The side-view imaging mode of radar is more susceptible to the influence of terrain inclination and data holes are easily formed in terrain areas with large slope fluctuations, which greatly affects the data accuracy [28,29]. Therefore, fully combining the respective advantages of multiple sources and effectively integrating them is an effective way to improve the quality of public DEM data [30,31]. In this study, three methods, GS fusion, feature-point-embedding fusion and partition-weighted fusion, were used to explore the mutual enhancement of the three data types. The three fusion methods of five plots were combined, the errors and extreme values of the errors before and after the fusion were compared and the application was analyzed [32]. The optimal algorithm for improving DEM accuracy in different terrains was achieved. A comparison of the fused-extracted river network with the 5 m DEM-extracted river network is shown in Figure 7. The statistical characteristics of errors before and after fusion are shown in Figure 8.

Through the analysis of the error statistical characteristics before and after the fusion, the accuracy of the original DEM data of the plot used in this study was generally found to have the best performance in the ALOS data, with SRTM data coming in second, and the ASTER data had the worst performance [33]. Additionally, as the average slope of the plot increases, the error and extreme error values in the original data show an increasing trend [34].

GS fusion has high fidelity. After fusion, it can not only maintain the advantages of the original data to a certain extent, but also achieve the effect of data mutual enhancement and improve data accuracy. The three combinations of GS fusion in this study all improved the accuracy of the original data, but the improvement effect of some plots was not evident and none of them improved by a whole level. The degree of accuracy improvement after fusion had a low correlation with the slope of the sample area and a greater correlation with the quality of the original data [35]; thus, the AS fusion result is the best overall.

Embedding fusion based on feature points can correct the large error values of ridges and valleys caused by factors such as shadows or vegetation. Feature-point-embedding fusion of the five plots in this study effectively improved the elevation RMSE and the extreme error value. Since the feature points come from the high-precision 5 m DEM basic data, the correction effect on the original data was greater, the accuracy improvement effect was evident and the accuracy was improved by one level. The accuracy of some plots was not improved by a level and the RMSE was also significantly reduced, approaching the critical value. The original data quality is still the most relevant to the improvement in the accuracy of the feature-point-embedding and fusion process, so ALOS data fusion is generally used to obtain the most ideal results. However, although the original poor-quality data could not achieve the best results, the degree of accuracy improvement was even greater. The error eigenvalue effect of each plot of the ASTER data was the worst, but the improvement effect of the fusion was the most significant.

The weight-based multi-source DEM data fusion method takes into account the different accuracies of DEM data from different sources and the fusion process is likewise adjusted, leading to different results. An appropriate weight allows the fusion to reach the best fusion ratio, so as to achieve the best result [12]. Partition-weighted fusion better considers the difference in the DEM accuracy of different slopes in complex terrain. In this study, the overall data accuracy of the partition-weighted fusion was improved significantly [36]. After the division, the degree of accuracy improvement of different slopes was significantly different. The error improvement degree was best in the flat area at the bottom of the ditch, followed by the flat area between the ditch, and the worst value was found in the steep area of the ditch slope. After the fusion of flat areas, the accuracy of the results will be increased by one level or decreased significantly closer to the lower index value. The accuracy in steep areas will not increase by one level, but it will be close to the low index value. The best smooth regional fusion effect was achieved with Shenmu AS fusion, Chunhua AA fusion, Ganquan AS fusion, Suide AS fusion and Yanchuan AA fusion. The best fusion effect in steep areas was achieved with Shenmu SA fusion, Chunhua SA fusion, Ganquan AA fusion, Suide AA fusion and Yanchuan AA fusion. In general, it can be concluded that AS fusion leads to a better fusion effect when the terrain is relatively gentle. AA fusion exerts a greater advantage in areas with moderately undulating terrain. The overall AA fusion accuracy improved the most. When SA was fused, a relatively stable range of improvement could be achieved more often, and there were no significant differences in the accuracy of the improvement. Among the three combination methods, that with the greatest impact on the accuracy improvement was the partition slope, followed by the quality of the original data.

## 5. Conclusions

The DEM accuracy was improved by the three methods. The precision of most data obtained via partition-weighted fusion and feature-embedding fusion can be improved by one grade or more. GS fusion accuracy was improved in a simple and convenient manner; in the feature-point-embedding fusion method, ALOS data obtained the best data results and ASTER data improved the accuracy most significantly. In partition-weighted fusion, AS fusion was applicable to the gentle region, while AA fusion was applicable to the steep region.

In this study, five typical gully areas in the Loess Plateau region were selected as sample areas to analyze applicable fusion methods for DEM accuracy enhancement. However, to further investigate the response pattern of error and topographic factors, more sample study areas need to be studied. In the future, more fusion methods will be applied to establish fusion models applicable to different geomorphic areas according to the statistical parameters (topographic factors) of each sample area. What is more, this study uses what is called the white box method. In this era of machine learning and artificial intelligence technologies, how to apply machine learning methods to this research remains to be explored.

## Figures and Tables

**Table 1:** The terrain of the study area.

	Gully Density (km/km^2^)	Slope (°)	Average Slope (°)	Elevation (m)	Relative Difference (m)
Shenmu	3.40	0–61.11	8.75	1197.84	286.50
Suide	6.52	0–79.46	28.55	997.44	373.66
Yanchuan	6.78	0–83.05	30.80	1090.28	327.24
Ganquan	5.60	0–76.16	26.47	1300.56	311.18
Chunhua	3.13	0–72.62	11.96	1045.79	388.86

**Table 2:** ALOS, SRTM and ASTER data characteristics.

Data Type	ALOS	SRTM	ASTER
Plane precision	2.5 m	20 m	30 m
Elevation accuracy	12.5 m	16 m	20 m
Spatial resolution	12.5 m	30 m	30 m
Data sources	http://www.eorc.jaxa.jp/ALOS/en/aw3d30/index.htm (accessed on 10 April 2020)	http://srtm.csi.cgiar.org (accessed on 10 April 2020)	http://www.gscloud.cn (accessed on 10 April 2020)

**Table 3:** The partition fusion weight table of various areas.

		Area Proportion	AS	AA	SA
Shenmu	Flat area at the gully bottom	19%	0.552	0.806	0.759
	Flat area between gullies	37%	0.791	0.979	0.800
	Gully slope steep area	44%	0.217	0.484	0.692
Yanchuan	Flat area at the gully bottom	28%	0.553	0.584	0.573
	Flat area between gullies	15%	0.533	0.544	0.546
	Gully slope steep area	57%	0.233	0.471	0.618
Chunhua	Flat area at the gully bottom	14%	0.706	0.886	0.927
	Flat area between gullies	25%	1.000	0.875	0.840
	Gully slope steep area	61%	0.422	0.722	0.808
Ganquan	Flat area at the gully bottom	11%	1.000	1.000	0.699
	Flat area between gullies	24%	0.966	0.888	0.491
	Gully slope steep area	65%	0.919	0.889	0.614
Suide	Flat area at the gully bottom	12%	1.000	0.904	0.473
	Flat area between gullies	18%	1.000	0.890	0.599
	Gully slope steep area	70%	0.966	0.788	0.526

**Table 4:** 1:50,000 DEM accuracy standard.

Terrain Category	Ground Slope (Unit: °)	Grid Point Elevation Error (Unit: m)
Flat ground	<2	4
Hilly	2–6	7
Mountain	6–25	11
Alpine mountain	>25	19

**Table 5:** Error statistic parameters in different study areas obtained via ENVI GS fusion (unit: m).

		ALOS	SRTM	ASTER	AS	AA	SA
Shenmu	R	7.683	6.924	8.804	6.830	8.278	7.592
	Rmax	33.000	29.000	46.000	27.000	38.000	32.000
	Rmin	−32.000	−24.000	−35.000	−25.000	−28.000	−21.000
Chunhua	R	12.117	12.257	19.947	12.062	13.570	13.699
	Rmax	44.000	45.000	110.000	44.000	50.000	50.000
	Rmin	−56.000	−57.000	−90.000	−56.000	−50.000	−49.000
Ganquan	R	8.123	13.625	15.456	8.137	9.775	14.965
	Rmax	33.000	48.000	77.000	32.000	28.000	45.000
	Rmin	−42.000	−50.000	−90.000	−42.000	−46.000	−54.000
Suide	R	9.958	15.523	16.238	9.849	10.808	15.858
	Rmax	33.000	29.000	46.000	27.000	38.000	32.000
	Rmin	−36.000	−57.000	−86.000	−25.000	−28.000	−21.000
Yanchuan	R	17.225	15.764	17.559	16.006	17.107	15.888
	Rmax	78.000	80.000	88.000	80.000	78.000	81.000
	Rmin	−62.000	−62.000	−93.000	−63.000	−62.000	−62.000

Note: AS indicates the ALOS and SRTM data fusion result, AA indicates the ALOS and ASTER data fusion result, SA indicates the SRTM and ASTER data fusion result, R indicates RMSE (root mean square error), Rmax indicates the maximum value of the error and Rmin indicates the minimum value of the error. The same below.

**Table 6:** Error statistical values before and after embedding feature points (unit: m).

		ALOS (Before/After Fusion)		SRTM (Before/After Fusion)		ASTER (Before/After Fusion)	
Shenmu	R	7.683	5.135	6.924	5.216	8.804	5.950
	Rmax	33.000	30.230	29.000	28.810	46.000	37.830
	Rmin	−32.000	−23.560	−24.000	−25.220	−35.000	−20.180
Suide	R	9.958	8.550	15.523	9.299	16.238	9.507
	Rmax	71.000	53.035	81.000	54.332	82.000	61.412
	Rmin	−36.000	−32.046	−51.000	−42.950	−86.000	−31.330
Yanchuan	R	17.225	11.304	15.764	10.357	17.559	10.390
	Rmax	78.000	54.660	80.000	49.670	88.000	51.100
	Rmin	−62.000	−58.170	−62.000	−57.710	−93.000	−55.290
Chunhua	R	12.117	9.937	12.257	10.011	19.947	12.131
	Rmax	44.000	38.424	45.000	38.880	110.000	77.778
	Rmin	−56.000	−37.110	−57.000	−33.590	−90.000	−36.970
Ganquan	R	8.123	8.277	13.625	9.627	15.456	9.438
	Rmax	33.000	36.020	48.000	36.790	77.000	48.430
	Rmin	−42.000	−29.020	−50.000	−35.400	−90.000	−44.840

**Table 7:** The error statistical value of partition-weighted fusion in Shenmu (unit: m).

		ALOS	SRTM	ASTER	AS	AA	SA
Flat area at the gully bottom	R	6.324	6.510	8.111	4.905	5.100	5.272
	Rmax	23.000	29.000	33.000	18.792	18.806	21.000
	Rmin	−32.000	−28.000	−38.000	−18.624	−22.358	−18.579
Flat area between gullies	R	4.369	5.449	7.816	3.775	3.888	4.790
	Rmax	31.000	29.000	35.000	30.373	30.874	27.400
	Rmin	−14.000	−12.000	−14.000	−9.995	−10.874	−8.000
Gully slope steep area	R	9.877	8.243	9.762	7.789	8.602	7.540
	Rmax	29.000	29.000	42.000	25.434	32.804	28.080
	Rmin	−35.000	−34.000	−31.000	−28.170	−33.452	−26.308

**Table 8:** The error statistical value of partition-weighted fusion in Yanchuan (unit: m).

		ALOS	SRTM	ASTER	AS	AA	SA
Flat area at the gully bottom	R	15.937	16.572	18.354	13.726	13.298	15.311
	Rmax	58.000	51.000	88.000	43.325	49.105	57.124
	Rmin	−41.000	−33.000	−44.000	−23.848	−27.27	−27.988
Flat area between gullies	R	15.162	15.451	16.239	12.465	11.154	12.943
	Rmax	49.000	39.000	57.000	28.137	26.145	27.184
	Rmin	−59.000	−62.000	−93.000	−47.467	−55.85	−57.884
Gully slope steep area	R	18.348	15.454	17.541	14.191	13.421	13.554
	Rmax	78.000	80.000	77.000	77.233	76.942	76.617
	Rmin	−62.000	−59.000	−89.000	−56.136	−61.805	−61.405

**Table 9:** The error statistical value of partition-weighted fusion in Chunhua (unit: m).

		ALOS	SRTM	ASTER	AS	AA	SA
Flat area at the gully bottom	R	10.033	11.074	18.473	10.682	10.483	10.992
	Rmax	39.000	33.000	89.000	33.058	33.72	36.759
	Rmin	−36.000	−45.000	−87.000	−39.764	−38.114	−44.394
Flat area between gullies	R	7.477	9.529	18.482	8.532	8.46	10.482
	Rmax	36.000	42.000	72.000	37.000	35.625	41.400
	Rmin	−29.000	−21.000	−42.000	−30.000	−27.500	−22.520
Gully slope steep area	R	13.894	13.410	20.836	15.214	14.395	14.267
	Rmax	44.000	45.000	110.000	48.266	47.610	45.504
	Rmin	−56.000	−57.000	−90.000	−49.532	−52.170	−46.616

**Table 10:** The error statistical value of partition weighted fusion in Ganquan (unit: m).

		ALOS	SRTM	ASTER	AS	AA	SA
Flat area at the gully bottom	R	7.834	14.169	17.143	7.784	7.784	14.593
	Rmax	27.000	37.000	51.000	21.000	21.000	25.087
	Rmin	−32.000	−48.000	−87.000	−31.000	−31.000	−47.321
Flat area between gullies	R	5.893	11.430	11.287	5.875	5.794	8.857
	Rmax	36.000	39.000	77.000	24.868	25.672	41.648
	Rmin	−26.000	−28.000	−46.000	−20.132	−19.672	−31.054
Gully slope steep area	R	9.574	14.858	16.760	9.821	9.805	14.119
	Rmax	31.000	40.000	63.000	26.676	25.540	33.760
	Rmin	−36.000	−50.000	−81.000	−44.704	−42.920	−50.770

**Table 11:** The error statistical value of partition weighted fusion in Suide (unit: m).

		ALOS	SRTM	ASTER	AS	AA	SA
Flat area at the gully bottom	R	8.290	12.249	13.731	6.432	12.132	11.088
	Rmax	29.000	28.000	70.000	28.000	36.662	33.836
	Rmin	−36.000	−54.000	−86.000	−25.000	−15.408	−62.823
Flat area between gullies	R	9.772	16.840	18.708	7.923	8.450	15.838
	Rmax	46.000	53.000	82.000	36.000	36.927	44.250
	Rmin	−22.000	−33.000	−29.000	−16.000	−14.043	−19.188
Gully slope steep area	R	10.797	15.154	15.489	9.994	9.800	13.022
	Rmax	71.000	81.000	79.000	61.340	63.756	72.422
	Rmin	−36.000	−57.000	−82.000	−37.170	−34.244	−47.492

## Data Availability

The data presented in this study are available on request from the corresponding author.
